# What Do Your Neighbors Think About You? How Perceived Neighbor Attitudes Toward Latinos Influence Mental Health Among a Pregnant Latina Cohort

**DOI:** 10.1007/s40615-023-01684-5

**Published:** 2023-06-30

**Authors:** Kristine J. Chua, Delaney A. Knorr, Janelly Jimenez, Arlene Francia, Valeria Rojas, Jhoana Infante Garcia, Molly Fox

**Affiliations:** 1 Department of Anthropology, University of California Los Angeles, 341 Haines Hall, 375 Portola Plaza, Los Angeles, CA 90095, USA; 2 California Center for Population Research, University of California Los Angeles, Los Angeles, CA 90095, USA; 3 Department of Psychiatry & Biobehavioral Sciences, University of California Los Angeles, Los Angeles, CA 90095, USA

**Keywords:** Neighborhood, Perceptions, Latino, Mental Health, Pregnancy

## Abstract

Latina women living in the USA experience disproportionately higher rates of psychological distress compared to their non-Latina White counterparts. Poor maternal mental health during pregnancy can contribute to intergenerational mental health disparities. Through this pathway, mothers’ experiences, environments, and exposures (henceforth “exposures”) during pregnancy become biologically embodied and can negatively affect the fetus and life-long developmental trajectories of her child. One of the exposures that can affect mother–offspring dyads is the neighborhood. With the goal of integrating anthropological and sociological theories to explain mental health disparities among pregnant Latina women, we explored how perceptions of neighbor attitudes may influence mental health during pregnancy. We analyzed self-reported responses from 239 pregnant Latina women in Southern California (131 foreign-born, 108 US-born) on their mental health and perceived attitudes of their neighbors using multiple linear regression models. Among foreign-born Latina women, living in neighborhoods with more favorable views of Latinos was associated with lower depression scores (pooled *β* = − .70, *SE* = .29, *p* = .019) and lower pregnancy-related anxiety scores (pooled *β* = − .11, *SE* = .05, *p* = .021), but greater state anxiety scores (pooled *β* = .09, *SE* = .04, *p* = .021). Among US-born women, there were no associations between neighbor attitudes and mental health. Overall, results suggest that social environments are correlated with mental health and that foreign-born and US-born Latinas have varied mental health experiences in the USA. Our findings highlight the importance of improving aspects of neighborhood cohesion as part of maternal–fetal care management.

## Introduction

Pregnancy is a particularly sensitive time for mother–offspring health. For the mother, there is a high risk for the onset of psychological distress, including depression, state anxiety, and pregnancy-related anxiety [1–3]. Poor maternal mental health can result in downstream consequences for fetal development and infant health, including preterm birth, low-birth weight, cognitive developmental deficits, and life-long non-communicable chronic disease risk [4–13]. Thus, investigating maternal mental health during pregnancy can have intergenerational public health implications.

The ecosocial model of health describes how distinct layers of social experiences can create population-level patterns of health disparities [14, 15]. Furthermore, people’s disparate experiences within a landscape of inequality can become embodied or “get under the skin” to expand minority health disparities [16, 17], which can have lasting intergenerational health effects [18]. Each of these interconnected levels of influence described by the ecosocial model can suggest strategies for intervention.

While community-level relationships, e.g., neighborhoods, have been studied extensively, much work has focused on poverty and deprivation [19–25]. Poverty and deprivation are significant points of influence, but studying only these issues limits our understanding of how a community functions. It is important to investigate the complex issues that prevent a community from thriving to create potential interventions toward improving the mental and physical health of individuals within said community. We make a unique contribution by expanding these questions of community-level impacts on mental health beyond only poverty or ethnic composition. Instead, we ask how a woman’s perceptions of the attitudes of her neighbors’ attitudes (henceforth “neighbor attitudes”) influence her mental health.

Subjective measures are critical here as there is growing evidence to suggest that perceptions over actual neighborhood characteristics may impose greater influence on mental health, particularly in minoritized families [22, 26–30]. In other work characterizing Latino mental health, scholars have shown that within Mexican American mother–offspring dyads, the subjective neighborhood, or how mothers perceive their neighborhoods and neighbors’ attitudes to them, relayed valuable information regarding their acceptance of them and their culture [29, 31]. Other aspects of neighborhoods, including cohesion and sociality, have also been shown to positively impact residents’ mental health [29, 32]. Together, these studies underscore the importance of considering subjective neighborhood and neighbor measures and their implications for health outcomes.

### Latino Mental Health

Mental health disparities among racial and ethnic minority communities in the USA is a well-established issue. We focus on Latino mental health in the USA for several reasons. First, Latinos are one of the fastest-growing and largest ethnic minority groups in the USA (now second behind Asian Americans) [33]. Latinos tend to be disproportionately affected by various systemic, social, cultural, and economic barriers in the USA [34], which contributes to worse mental health outcomes [35]. Additionally, stressors are shown to contribute cumulatively to worsened mental health among reproductive-aged women, rather than a sensitized or habituated response [36]. The myriad of stressors affecting Latinos in the USA widens the minority mental health disparities in the USA. In parts of Los Angeles County, Latina mothers are most likely (25.9%) to be uninsured 6 months before becoming pregnant, as well as experience greater rates of fertility, preterm births, and low birthweights compared to mothers of other ethnic and racial groups [37]. Lastly, the Latino community faces many structural and cultural barriers that prevent them from receiving mental health treatment. Structural barriers include lack of health insurance or cultural miscommunication in health care, while cultural barriers include mental health stigma [38–40]. Only 31% of Latino adults with mental health disorders receive treatment each year compared to the USA, average of 45% [41]. Latina women experience rates of psychological distress 20% higher than non-Latina White women [42]. Latina women are also more likely to experience depressive symptoms, reportedly 27% higher compared to Latino men [39, 43]. These percentages vary by country of origin: US-born Latina women experience higher depression (44%) compared to foreign-born Latina women (34%) [44].

We focus on Latinos in Southern California because of its large Latino population compared to the rest of the USA (18.7% of the total population identifying as Latino in the USA compared to 40% of the total population in Southern California; [45]). Residents of Latin-American heritage represent 48% of the population in Los Angeles County and 34% of Orange County [45, 46]. In Los Angeles County, over one in three female residents are foreign-born, and of these residents, 56% are from Latin American countries [46].

### Neighborhood Theoretical Frameworks

Latino mental health has been considered through the lens of social disorganization theory. Social disorganization theory [47, 48] hypothesizes that the quality of a neighborhood depends on three factors: poverty, residential stability, and ethnic composition. With regard to ethnic composition, social disorganization theory hypothesizes that higher concentrations of Latinos (i.e., ethnic homogeneity) promote resident mental health due to shared experiences [49]. Ethnic homogeneity lends itself to enabling trust among neighbors and increased social capital, thereby benefiting residents [50]. Consistent with the social disorganization theory, Latinos living in homogenous neighborhoods tend to have more social cohesion [32], better adolescent mental health [49, 51], and prenatal mental health [31] compared to ethnically heterogeneous neighborhoods. Notably, Curci and colleagues [31] found that a higher Latino neighborhood concentration promotes positive maternal mental health outcomes and child health outcomes among 322 Mexican American mother–offspring dyads from low-income neighborhoods in Phoenix, Arizona. While work from social disorganization theory has been influential in understanding Latino mental health, empirical evidence supporting this hypothesis is mixed [52]. White and colleagues [49], for instance, recently conducted a meta-analysis and found that children residing in less concentrated Latino neighborhoods actually fared better and had fewer mental health issues, contrary to the predictions of social disorganization theory. Thus, previous literature has inconsistencies regarding the relation between neighborhood ethnic composition and mental health among Latinos.

How these inconsistencies emerge stems partially from methodological and with it, conceptual issues pertaining to how homogenous neighborhoods are being measured. A large focus has been on constructs like foreign-born concentration, indexed by a composite percentage of Latino residents, or racial-ethnic concentration, indexed by the percent of Latino residents. These are imperfect assessments because these concentration indices could represent either homogeneous or heterogeneous neighborhoods as these estimates fail to account for factors like country of origin and geographic sampling bias [52]. The use of these constrained metrics likely stems from the gross unilateral categorizations that are often imposed on racial and ethnic minorities in the USA [53]. Latinos fall outside of the US system of racial categorization. Despite many health and population researchers acknowledging this limitation, there is no clear consensus or way forward for replacing this system of racial categorization.

Moreover, previous studies have conflicting results because neighborhood poverty and Latino concentration are highly correlated [52]. This is in part due to structural issues like redlining, gentrification, and/or racism that tend to systematically assort individuals into neighborhoods and thus create homogeneous neighborhoods. Refinements to social disorganization theory have been suggested by Kubrin and Weitzer [54] and White et al. [49, 52]. Notable suggestions include refraining from the use of concentration indices; increasing attention to the potential heterogeneity within a given group; and for the use of more culturally appropriate datasets that focus on the individual in order to capture more nuance.

Emerging work from segmented assimilation theory overlaps with social disorganization theory, in that higher concentrations of Latinos in a given neighborhood lend themselves to be “socially organized” due to a shared set of similar values, morals, and culture that creates relatively stable social cohesion. This tends to result in segmented groups that may not be a part of the hegemonic cultural group [49, 55]. Thus, there is less reliance on the socio-economic class and position of the residents and a higher emphasis on creating social structures and capital that allow its members to prosper. Importantly, segmented assimilation theory offers a more nuanced perspective in that they account for within-group cohesion, the idea that established groups might be able to offer benefits that are specific to their within-group members. It also recognizes that socially organized neighborhoods (i.e., established groups) can co-occur with other systemic challenges felt by the community, e.g., discrimination; [25, 52]. From this perspective, it acknowledges the fact that neighborhoods with high ethnic concentrations may be faced with higher forms of oppression, but that its residents may have developed behavioral strategies, e.g., disengagement, to mitigate the potential downsides of residing in these neighborhoods [52]. In addition, segmented assimilation theory recognizes how ethnic homogeneity can have negative downstream influences that can result in culture clashes between generations [55]. This is particularly salient when there are differences in expectations driven by societal and cultural norms (e.g., gender, family structure). Within this framework exists a stronger emphasis on relationships between neighbors, like shared values and social support, e.g., [56]. Focusing on these shared values can help encourage and support new forms of social capital that is needed for Latino communities to thrive.

### Current Study

In the current study, we adopt a segmented assimilation perspective, which combined with the ecosocial model of health, can inform our understanding of reproductive and offspring health. The goal of this study is to address how a woman’s perceptions of her neighbors’ attitudes toward Latinos influence prenatal mental health during pregnancy. Through this framing, we aim to examine neighborhood quality in a way that goes beyond that of poverty and of ethnic composition. Specifically, we evaluate the relationship between perceptions of neighbor attitudes toward Latina culture and mental health among pregnant Latina women residing in Southern California. We focus on subjective measures of neighbor attitudes toward Latinos as this reflects how Latina mothers’ mental health might be influenced by the sociocultural environment of their neighborhood. We stratify our analyses by country of birth since previous work has shown different rates of affective disorders and mental health resources available to foreign-born and US-born women [57]. We predict that women who perceive their neighbors to be less accepting of them and their culture will experience greater levels of depression, state anxiety, and pregnancy-related anxiety as these feelings of rejection and low social support may exacerbate poor mental health. We are agnostic as to whether there will be any meaningful differences based on foreign-born status.

## Methods

### Cohort

This study is part of a larger research program, the Mothers’ Cultural Experiences (MCE) project, which aims to understand the health effects of maternal sociocultural experiences, and how those health effects are potentially transmitted to offspring during prenatal and early postnatal life. Eligible women were 18 years or older, fluent in either English or Spanish, and self-identified as Latina, Hispanic, Chicana, Mexican, and/or Latin American.

Data from an initial Wave 1 cohort, collected from 2016 to 2018 consisted of 361 pregnant and postpartum women recruited from medical clinics and breastfeeding classes in Los Angeles and Orange counties of Southern California. Thirteen women were deemed ineligible after the study ended. We omitted an additional 103 postpartum women because of our focus on the prenatal period and an additional nine women who did not indicate whether they were foreign-born or US-born, which was the criterion used to split the cohort. Thus, our final analytic cohort consisted of 239 women.

### Material and Procedure

All materials and procedures were approved by the Institutional Review Boards of all participating institutions with appropriate reliances. This study adheres to the tenets of the Declaration of Helsinki. Women provided self-report data on their mental health, demographics, neighborhood perceptions, and their cultural orientation. We compensated them following the completion of the questionnaire, which was presented in either Spanish or English.

### Variable Operationalization

#### Predictor Variable: Neighbor Attitudes

We utilized the Neighborhood Attitudes Toward Latinos scale [58] to determine the perceptions women have of their neighbors’ attitudes toward Latinos. This scale consisted of six items measured on a 5-point Likert scale (0 = Not at all true to 4 = Very true; e.g., “*People in this neighborhood appreciate Latino culture and people”).* Higher values on this scale indicate greater perceived neighbor acceptance toward Latino culture. We found that the reliability of this scale was low in our sample (Cronbach’s *ɑ* = 0.45). After conducting a confirmatory factor analysis, we found that the reliability model produced an adequate fit, *χ*^2^(5) = 10.79, *p* = 0.06; CFI = 0.97; TLI = 0.94, RMSEA = 0.08. However, in both the confirmatory factor analysis and when investigating the measure’s internal reliability, we found that the reverse-scored items did not significantly load onto the factor (Supplementary Materials). Thus, we removed the two reverse-coded items, and the resulting scale comprised four items, which exhibited better reliability (*ɑ* = 0.80). The Cronbach’s alpha for this modified scale was 0.83 for women taking the survey in English (henceforth *ɑ_E_*) and 0.76 for women taking the survey in Spanish (henceforth *ɑ_S_*).

#### Outcome Variables: Mental Health

We measure three mental health outcomes (depression, state anxiety, and pregnancy-related anxiety) with validated psychometric scales. For depression, we used the Edinburgh Postnatal Depression Scale (EPDS) [59, 60], which calculated depression scores based on 10 items. Each item was ranked on a 4-point Likert scale, and the sum was taken for a final score (see Supplementary Materials for more details). We found high reliability with this scale (overall *ɑ* = 0.84; *ɑ_E_* = 0.86 and *ɑ_S_* = 0.82). For anxiety, we used the Spielberger State-Trait Anxiety Inventory State Short-Form scale (STAI-SF) [61]. The STAI-SF consists of six items, three of which are reversed coded, and anchored on a 4-point scale (1 = Not at all to 4 = Very much). STAI-SF scores were averaged, and the complete range of possible scores was 1–4 (for an item breakdown, see Supplementary Materials). Again, we found good reliability (overall *ɑ* = 0.81; *ɑ_E_* = 0.83 and *ɑ_S_* = 0.77). For Pregnancy-Related Anxiety scale (PRA) [62, 63], each of the ten items was scored from 1to 4 and then the average taken (overall *ɑ* = 0.89; *ɑ_E_* = 0.89 and *ɑ_S_* = 0.90). We also intended to use the Perceived Stress Scale (PSS) as another mental health outcome variable, but due to low reliability (overall *ɑ* = 0.50) decided to forgo these analyses.

We treated the mental health variables as continuous in all models. This was due to Bowins’ [64] argument describing how mental health variables like depression tended to exist as a continuum in nature as opposed to discrete categories. We presented descriptive summary metrics of all mental health variables and the percentages of those with clinically significant symptoms of depression for each subset of the cohort (Table 1).

#### Control Variables

In order to isolate the outcome variables of interest (i.e., depression, state anxiety, and pregnancy-related anxiety), we controlled for the other two mental health variables in the models (e.g., pregnancy-related anxiety models controlled for state anxiety and depression). We felt this was important since depression and anxiety comorbidities are common [65].

We also controlled for socio-economic status (SES) using a composite variable of subjective SES, education, and food insecurity developed by Fox [66]. This composite variable is made by standardizing each variable on a scale of 0–1 and then summing the three variables. This variable measures SES with the MacArthur Subjective Socioeconomic Status Scale [67], education with a single question of the highest level of education attained (i.e., “*What is your highest level of education?”),* and food insecurity with the two-item screen to identify families at risk for food insecurity [68]. We also controlled for relationship status and trimester in all models. These control variables each consistently affect prenatal mental health [8, 69], as well as neighbor attitudes [70].

### Analytic Strategy

#### Missingness and Imputation

There was a total of 5% missingness across all variables. In order to increase the efficiency of our estimates, we ran multiple imputations by chained equations (MICE) [71] on our final analytic cohort (*N* = 239) and produced five imputed datasets. We tested the difference in missingness with relevant variables of interest, such as depression and foreign-born, and found nothing falsifying our missing at-random assumption (Supplementary Materials).

#### Models, Regression Diagnostics, and Data Analysis

We ran multiple linear regression models on all five imputed datasets. We performed Breusch-Pagan tests iteratively on each model among each of the five datasets. Depression and pregnancy-related anxiety models were consistently rejected by the Breusch-Pagan test, indicating heteroskedasticity in the data. As such, robust standard errors were calculated for all models. Variance inflation factor calculations also ensured low levels of multicollinearity in the models (Supplementary Materials).

We examined the effects of neighborhood attitudes of Latinos in three separate models, one for each mental health outcome, controlling for socio-economic status, relationship status, trimester, and other mental health variables. We ran the model separately for foreign-born and US-born women. All statistical analyses were performed in R statistical software, v4.0.3. We present pooled results below.

## Results

### Demographics

The first subset of the analytic cohort was composed of 108 foreign-born women (*M*_age_ = 30.90, *SD* = 1.21) who were primarily born in Mexico (78.6%), in relationships (89%), and experiencing high rates of food insecurity (40.5%). Using the clinical EPDS cutoff score for depression (score > 10), 16.8% of foreign-born women in our cohort had a high likelihood of at least minor clinical depression.

The second subset of the analytic cohort was composed of 131 US-born women (*M*_age_ = 27.70, *SD* = 5.51), also predominantly in relationships (87%) and experiencing high rates of food insecurity (38.0%). Using the clinical EPDS cutoff, 15.7% of US-born women in our cohort had a high likelihood of at least minor clinical depression. US-born and foreign-born women did not have statistically different mental health scores but had significantly different ages (Table 1).

Most women reported a large presence of Latinos in their neighborhoods (25.9% of women reported about half of their neighbors are Latino, and 45.2% of women reported more than half of their neighbors are Latino; Table 1). Only 21% of women in our cohort reported Latinos made up less than half of their neighborhood (Table 1). While there was some variation, these neighborhood trends are consistent with demographic patterns found in Los Angeles and Orange counties.

Results revealed that among foreign-born women, those who viewed their neighbors as having more positive attitudes toward Latino culture had significantly lower depression (pooled *β* = − 0.70, *SE* = 0.29, *p* = 0.019) and lower PRA scores (pooled *β* = − 0.11, *SE* = 0.05, *p* = 0.021), and greater state-anxiety scores (pooled *β* = 0.09, *SE* = 0.04, *p* = 0.021) (Table 2). There were no significant associations with any of the mental health outcomes among US-born women (Table 2).

Model comparison calculated from five imputed data sets against their respective null models produced the following pooled values among the foreign-born cohort (F-statistics; *p* values): EPDS (14.73; < 0.0001), STAI (12.87; < 0.0001), PRA (4.58; < 0.0001) and among the US-born cohort: EPDS (22.46; < 0.0001), STAI (19.91 < 0.0001), PRA (7.39; < 0.0001).

## Discussion

We seek to integrate anthropological and sociological theories to explain mental health disparities among pregnant Latina women. The many interconnected levels of influence described by the ecosocial model suggest that health is not just our biology, but everything about the individual’s physical and socio-cultural environment. One of the exposures that can contribute to mother–offspring dyads is the role of neighborhoods. Thus, we find that a segmented assimilation perspective complemented the ecosocial model of health in that it provided a more nuanced application for measuring and accounting for neighborhood dynamics. Our focus on neighbor attitudes and their associations with maternal psychological distress is a first step to understanding intergenerational health among pregnant Latina women.

Our models found that foreign-born women who perceived their neighbors to have more positive attitudes toward Latinos tended to have lower levels of depression and pregnancy-related anxiety, but higher levels of state anxiety. That is, greater neighborhood acceptance of Latino people, language, or culture was associated with reduced psychological distress in two domains, and higher in one domain, only for our foreign-born cohort subset. These results are relatively consistent with other work demonstrating how neighborhood characteristics can negatively influence prenatal mental health [16, 31, 72, 73].

One possible explanation for the relationship between positive neighbor attitudes and better mental health may be attributed to the benefits of a positive social network. Among pregnant women living in Spain, social cohesion positively influenced mental health among only low and medium-SES groups [74]. This may be due to higher population density and the physical space, which lends itself to frequent interactions. Another possibility is that these women may reside in a neighborhood where their ethnic culture is preserved [75], resulting in a neighborhood that by default, will have a more positive view of Latinos. This harkens back to much of the discussed work on ethnic composition and social cohesion. However, van der Meer and Tolsma’s [76] review of this work does not find associations of ethnic diversity and interethnic social cohesion. Furthermore, there have been recent calls to re-evaluate how neighborhoods and ethnic concentrations are measured [53, 77]. Past studies tend to rely on the U.S. Census data, which often portrays an inaccurate assessment of racial/ethnic density. One of the main issues stems from the deeply rooted colonial narratives that treat ethnic and racialized minorities as discrete entities, White versus non-White. Additional confusion exists for Latinos who often feel unrepresented by the categories provided by the U.S. Census [53]. Thayer and colleagues [75] recommend the use of integrating both perceived and objective measures to assess neighborhood quality, which can be used in future research when examining neighborhood quality, social cohesion, and socio-cultural space.

It is important to note that state anxiety runs counter to our expected results and is inconsistent with previous work. It is possible that issues with the measure itself could have contributed to the mixed findings. Despite generally positive psychometric features of the STAI, scholars have criticized its use due to its inability to capture “pure anxiety” that is distinguishable from depression, thus, calling for better measures that accurately capture anxiety and its symptomatology [78]. Increased state anxiety among foreign-born women can signify challenges to conform to social norms within the neighborhood. Another plausible explanation for our findings that is unrelated to measurement concerns may stem from the pressure to acculturate and assimilate to the USA, e.g., [18, 79]. Statistical limitations related to power or confounding may also be responsible for unexpected findings (see Limitations below).

Our results revealed substantial differences between US-born and foreign-born women with regard to how perceptions of neighbors’ attitudes relate to prenatal mental health. Previous research highlights differences between US-born and foreign-born Latinos, including differences in social support networks, cultural values, mental health, and reasons for living in ethnic enclaves [39, 55, 57, 80]. These differences motivated us to separate the cohort by country of birth. However, we cannot say definitively why the results for US-born women were null. This may be due to the differences in cohort sample size. Alternatively, there may be distinct features about the US-born cohort, such as them being less sensitive to neighbor attitudes, but we will not speculate further.

We contribute to the literature by integrating a confluence of theoretical frameworks from anthropology, sociology, and ethnic studies to address the importance of neighbor attitudes on pregnant Latina mental health. We seek to move beyond strictly focusing on the ethnic composition of an individual’s neighbors. Additionally, it is critical to intergenerational health to ask these questions during pregnancy, thereby blending health disparity and sociology research frameworks. If neighbor attitudes do indeed play a role in pregnant Latina mental health, then this work could complement existing literature, and future work might benefit from investigating the heterogeneity of perceptions, experiences, and cultures within neighborhoods. The ultimate goal is to mitigate mental health disparities and inequities among Latinos.

## Public Health Implications

Given the complexities of the incidence of depression and other mental health disorders within the Latino population, understanding the mechanism by which mental health disorders ensue in pregnant Latina women can help public health officials design interventions that target specific risk factors of mental health disorders. Prenatal maternal psychological distress (including stress, anxiety, and depression) has also been associated with low birth weight and pre-term birth [3, 9, 10, 81]. Low-birth weight and preterm birth are frequent causes of infant morbidity and mortality [82, 83], which in turn is implicated in the offspring’s long-term disease risk, including cardiovascular disease, obesity, diabetes, and psychopathology [82]. As a result, designing effective treatment and prevention plans for prenatal mental health is imperative within Latina communities.

Our findings suggest that public health interventions should promote diversity and cultural exchange at the neighborhood level in addition to improving aspects of neighborhood cohesion as part of maternal–fetal care management. We note here that increasing diversity does not necessarily mean that only members belonging to a racial or ethnic minority should be confined to a neighborhood. Rather, increasing diversity involves all members of a community and integrates various perspectives. To increase positive views of others and others’ cultures, one possible intervention could be to provide cultural education for the community [38]. Here, we underscore the importance of creating community cultural centers that can foster diversity, ethnic identity, and education programs. This type of intervention has the potential to reduce incidences of poor mental health among Latino adults and youth, cultivate coping skills for Latino adolescents who might face discrimination [44], and promote education for non-Latino community members who might be unfamiliar with Latino culture and values [38].

## Limitations

We cannot comment on causality due to the observational design of our cross-sectional study. While we suggest, for example, that negative neighbor attitudes of Latinos may result in poorer mental health outcomes, the opposite causal direction may also be true: poor mental health may lead women to view their neighbors’ perceptions of Latinos through a negative lens.

This study is only among pregnant women. While women experience other pregnancy-related stressors apart from neighborhood strain, we can only statistically control for stressors that may confound the results. Modeling our predictions in cohorts of only US-born or foreign-born pregnant women helps control for the unique stressors of pregnancy and birthplace. We chose control variables that influence both neighbor attitudes and psychological distress. Our dataset is limited to the variables in the study. Future studies will benefit from larger datasets that include all possible variables.

Similarly, we did not have enough information about women’s mental healthcare access or whether they were already seeking treatment with a mental healthcare provider. It is possible that mental healthcare could influence the findings, possibly explaining some of the null results. Future research will also benefit from a comprehensive list of women’s mental health history, access to professional services, and treatment.

We ran a post-hoc sensitivity analysis for F-tests in G*Power (v3.1) to assess the strength of our regression models, setting our parameters to an alpha-error of 0.05. This revealed that our designed models can detect effect sizes (*f* ^2^) as small as 0.116 for foreign-born and 0.142 for US-born with 80% power. According to Cohen’s [84] guidelines where *f* ^2^ ≥ 0.02 is a small effect size and *f* ^2^ ≥ 0.15 is a medium effect size, we are powered to detect small effect sizes more so among the foreign-born cohort subset. The pattern of differences between our two cohorts may be attributable to these differences in sample sizes.

We also acknowledge potential issues with the term “Latina,” which inherently describes a very diverse population, including women of many different socio-cultural experiences and ethnicities. Our sample is mostly women who are of Mexican descent. Our findings, therefore, may have limited generalizability beyond pregnant Mexican American women living in Southern California.

## Conclusion

Our findings contribute to the resurgent interest in work investigating the importance of perception of neighborhood attitudes and mental health among pregnant Latina women. We integrate theories and perspectives from anthropology, sociology, and health disparities research to examine how the wider social environment can influence mental health, particularly for foreign-born women, which can have implications for intergenerational embodiment. Future studies are needed to evaluate how the neighborhood context can improve maternal–fetal care management.

## Supplementary Material

supplement

## Figures and Tables

**Table 1:** Demographic and descriptive statistics of all mental health variables for each cohort

	US-born (*N* = 108)	Foreign-born (*N* = 131)	Total (*N* = 239)
Age (years)			
Mean (SD)	27.7 (5.51)	30.9 (6.33)	29.5 (6.17)
Median [min, max]	27.2 [18.2, 42.0]	30.7 [18.1, 45.3]	29.1 [18.1, 45.3]
Missing	5 (4.6%)	6 (4.6%)	11 (4.6%)
Relationship status			
In a relationship	94 (87.0%)	116 (88.5%)	210 (87.9%)
Single	11 (10.2%)	11 (8.4%)	22 (9.2%)
Missing	3 (2.8%)	4 (3.1%)	7 (2.9%)
Parity			
Nulliparous	43 (39.8%)	41 (31.3%)	84 (35.1%)
Parous	26 (24.1%)	37 (28.2%)	63 (26.4%)
Missing	39 (36.1%)	53 (40.5%)	92 (38.5%)
Education			
Less than high school	4 (3.7%)	27 (20.6%)	31 (13.0%)
High school or equivalent	67 (62.0%)	55 (42.0%)	122 (51.0%)
More than high school	35 (32.4%)	44 (33.6%)	79 (33.1%)
Missing	2 (1.9%)	5 (3.8%)	7 (2.9%)
Trimester			
First trimester	10 (9.3%)	8 (6.1%)	18 (7.5%)
Second trimester	23 (21.3%)	29 (22.1%)	52 (21.8%)
Third trimester	65 (60.2%)	80 (61.1%)	145 (60.7%)
Missing	10 (9.3%)	14 (10.7%)	24 (10.0%)
Food insecurity			
Yes	41 (38.0%)	53 (40.5%)	94 (39.3%)
No	55 (50.9%)	64 (48.9%)	119 (49.8%)
Missing	12 (11.1%)	14 (10.7%)	26 (10.9%)
Country of origin			
USA	108 (100%)	0 (0%)	108 (45.2%)
Mexico	0 (0%)	103 (78.6%)	103 (43.1%)
El Salvador	0 (0%)	10 (7.6%)	10 (4.2%)
Guatemala	0 (0%)	7 (5.3%)	7 (2.9%)
Another country	0 (0%)	6 (4.6%)	6 (2.5%)
Missing	0 (0%)	5 (3.8%)	5 (2.1%)
Depression (EPDS) (full scale range)			
Mean (SD)	5.46 (4.71)	5.70 (4.53)	5.59 (4.61)
Median [min, max]	5.00 [0, 23.0]	5.00 [0, 17.0]	5.00 [0, 23.0]
Missing	2 (1.9%)	6 (4.6%)	8 (3.3%)
Depression (EPDS)—clinically significant symptoms (> 10)			
Not depressed	89 (82.4%)	103 (78.6%)	192 (80.3%)
Depressed	17 (15.7%)	22 (16.8%)	39 (16.3%)
Missing	2 (1.9%)	6 (4.6%)	8 (3.3%)
State anxiety (full scale range)			
Mean (SD)	1.73 (.63)	1.63 (.53)	1.68 (.58)
Median [min, max]	1.67 [1.00, 4.00]	1.50 [1.00, 3.17]	1.50 [1.00, 4.00]
Missing	3 (2.8%)	8 (6.1%)	11 (4.6%)
Pregnancy-related anxiety (full scale range)			
Mean (SD)	1.61 (.53)	1.67 (.57)	1.64 (.55)
Median [min, max]	1.50 [1.00, 3.30]	1.50 [1.00, 3.75]	1.50 [1.00, 3.75]
Missing	2 (1.9%)	8 (6.1%)	10 (4.2%)
Number of Latino-identifying neighbors reported by participants			
About half the people	33 (30.6%)	29 (22.1%)	62 (25.9%)
Less than half	17 (15.7%)	33 (25.2%)	50 (20.9%)
More than half	50 (46.3%)	58 (44.3%)	108 (45.2%)
Missing	8 (7.4%)	11 (8.4%)	19 (7.9%)
Neighborhood attitudes toward Latinos scale			
Neighbors are mostly neutral about Latino culture in the community	35 (32.4%)	47 (35.9%)	82 (34.3%)
Neighbors are negative about Latino culture in the community	41 (38.0%)	43 (32.8%)	84 (35.1%)
Neighbors are positive about Latino culture in the community	24 (22.2%)	28 (21.4%)	52 (21.8%)
Missing	8 (7.4%)	13 (9.9%)	21 (8.8%)

**Table 2:** The relationship between social orientations and maternal mental health among foreign-born and US pregnant Latina

	Depression foreign-born	Depression US-born	State anxiety foreign-born	State anxiety US-born	Pregnancy-related anxiety foreign-born	Pregnancy-related anxiety US-born
Intercept	3.06	−6.35**	0.60*	1.40***	1.49***	1.39***
	(2.20)	(2.30)	(0.26)	(0.29)	(0.34)	(0.32)
Neighborhood attitudes of Latinos	−0.70*	−0.37	0.09*	−0.00	−0.11*	0.00
	–	–	–	–	–	–
	(0.30)	(0.27)	(0.04)	(0.04)	(0.05)	(0.04)
Socio-economic status	−0.60	−0.50	−0.08	−0.04	−0.05	−0.14
	(0.54)	(0.57)	(0.07)	(0.07)	(0.08)	(0.08)
Relationship status	−3.39**	−0.12	0.16	−0.09	−0.14	0.22
	(1.16)	(1.04)	(0.15)	(0.14)	(0.17)	(0.15)
Trimester	0.03	0.95*	0.02	−0.09	0.01	−0.10
	(0.54)	(0.48)	(0.07)	(0.07)	(0.09)	(0.07)
State anxiety	4.47***	4.70***	–	–	0.33**	0.16
	(0.63)	(0.57)			(0.11)	(0.11)
Pregnancy-related anxiety	0.40	1.74*	0.21**	0.14	–	–
	(0.61)	(0.69)	(0.07)	(0.10)		
Depression	–	–	0.06***	0.09***	0.01	0.04*
			(0.01)	(0.01)	(0.01)	(0.01)
*R* ^2^	0.44	0.59	0.43	0.56	0.20	0.33
*N*	131	108	131	108	131	108

*** *p* < 0.001;

** *p* < 0.01;

* *p* < 0.05.

*N*_foreign-born_ = 108. *N*_US-born_ = 131. Robust standard errors are in parenthesis. For foreign-born women, pooled, unadjusted-*R*^2^ = .44, 95% CI [.30, .57] for depression, .43 [.29, .55] for state anxiety, and .20, [.09, .34] for pregnancy-related anxiety, respectively. For US-born women, pooled, unadjusted-*R*^2^ = .59, 95% CI [.46, .70] for depression, .59, [.43, .68] for state anxiety, and .33, [.18, .47] for pregnancy-related anxiety, respectively. For adjusted-*R*^2^ run iteratively with each imputed dataset, see Supplementary Materials

## Data Availability

Not applicable.

## References

[1] Fishell A Depression and anxiety in pregnancy. J Popul Ther Clin Pharmacol. 2010;17(3):e363–9. 21041870

[2] Gurung R, Schetter C, Collins N, Rini C, Hobel C. Psychosocial predictors of prenatal anxiety. J Soc Clin Psychol. 2005;24:497–519. 10.1521/jscp.2005.24.4.497.

[3] Schetter CD, Tanner L. Anxiety, depression and stress in pregnancy: implications for mothers, children, research, and practice. Curr Opin Psychiatry. 2012;25(2):141. 22262028 10.1097/YCO.0b013e3283503680 PMC4447112

[4] Barker DJP. The origins of the developmental origins theory. J Intern Med. 2007;261(5):412–7. 10.1111/j.1365-2796.2007.01809.x. 17444880

[5] Bush NR, Effects of pre-and postnatal maternal stress on infant temperament and autonomic nervous system reactivity and regulation in a diverse, low-income population. Dev Psychopathol. 2017;29(05):1553–71. 10.1017/S0954579417001237. 29162167 PMC5726291

[6] Glover V Annual research review: prenatal stress and the origins of psychopathology: an evolutionary perspective. J Child Psychol Psychiatry. 2011;52(4):356–67. 10.1111/j.1469-7610.2011.02371.x. 21250994

[7] Gluckman PD and Hanson MA. “Developmental plasticity and the developmental origins of health and disease,” in Early Life Origins of Human Health and Disease, Newnham JP and Ross MG, Eds., Basel: KARGER, 2009;1–10. 10.1159/000221148.

[8] Glynn LM, Sandman CA. Prenatal origins of neurological development: a critical period for fetus and mother. Curr Dir Psychol Sci. 2011;20(6):384–9.

[9] Grigoriadis S, The impact of maternal depression during pregnancy on perinatal outcomes: a systematic review and meta-analysis. J Clin Psychiatry. 2013;74(4):8615. 10.4088/JCP.12r07968 23656857

[10] Grigoriadis S, Maternal anxiety during pregnancy and the association with adverse perinatal outcomes: systematic review and meta-analysis. J Clin Psychiatry. 2018;79(5):813. 10.4088/JCP.17r12011 30192449

[11] Kinsella MT, Monk C. Impact of maternal stress, depression & anxiety on fetal neurobehavioral development. Clin Obstet Gynecol. 2009;52(3):425. 19661759 10.1097/GRF.0b013e3181b52df1 PMC3710585

[12] Lewis AJ, Austin E, Knapp R, Vaiano T, Galbally M. Perinatal maternal mental health, fetal programming and child development. Healthcare. 2015;3(4):1212–27. 27417821 10.3390/healthcare3041212 PMC4934640

[13] Netsi E, Pearson RM, Murray L, Cooper P, Craske MG, Stein A. Association of persistent and severe postnatal depression with child outcomes. JAMA Psychiat. 2018;75(3):247–53. 10.1001/jamapsychiatry.2017.4363 PMC5885957 29387878

[14] Krieger N Theories for social epidemiology in the 21st century: an ecosocial perspective. Int J Epidemiol. 2001;30(4):668–77. 10.1093/ije/30.4.668. 11511581

[15] Krieger N Embodiment: a conceptual glossary for epidemiology. J Epidemiol Community Health. 2005;59(5):350–5. 10.1136/jech.2004.024562. 15831681 PMC1733093

[16] Conradt E, Carter SE, Crowell SE. Biological embedding of chronic stress across two generations within marginalized communities. Child Dev Perspect. 2020;14(4):208–14. 10.1111/cdep.12382. PMC12806165 41552782

[17] Gravlee CC. How race becomes biology: embodiment of social inequality. Am J Phys Anthropol. 2009;139(1):47–57. 10.1002/ajpa.20983. 19226645

[18] Fox M, Entringer S, Buss C, DeHaene J, Wadhwa PD. Intergenerational transmission of the effects of acculturation on health in Hispanic Americans: a fetal programming perspective. Am J Public Health. 2015;105(Suppl 3):S409–23. 10.2105/AJPH.2015.302571. 25905831 PMC4455494

[19] Chua KJ, Lukaszewski AW, Grant DM, Sng O. Human life history strategies: calibrated to external or internal cues? Evol Psychol. 2016;15(1):147470491667734. 10.1177/1474704916677342. PMC10638872 28164721

[20] Kaufman-Shriqui V, Neighbourhood-level deprivation indices and postpartum women’s health: results from the Community Child Health Network (CCHN) multi-site study. Health Qual Life Outcomes. 2020;18(1):38. 10.1186/s12955-020-1275-x. 32087734 PMC7036181

[21] Krieger N, Structural racism, historical redlining, and risk of preterm birth in New York City, 2013–2017. Am J Public Health. 2020;110(7):1046–53. 10.2105/AJPH.2020.305656. 32437270 PMC7287548

[22] Kruger DJ, Reischl TM, Gee GC. Neighborhood social conditions mediate the association between physical deterioration and mental health. Am J Community Psychol. 2007;40:261–71. 17924185 10.1007/s10464-007-9139-7

[23] Nettle D Dying young and living fast: variation in life history across English neighborhoods. Behav Ecol. 2010;21(2):387–95. 10.1093/beheco/arp202.

[24] Thayer ZM. Dark shadow of the long white cloud: neighborhood safety is associated with self-rated health and cortisol during pregnancy in Auckland, Aotearoa/New Zealand. SSM - Popul Health. 2017;3:75–80. 10.1016/j.ssmph.2016.11.004. 29349206 PMC5768991

[25] White RM, Kho C, Nair RL. US Mexican-origin adolescents’ well-being in the context of neighborhood White concentration. J Res Adolesc. 2022;32(2):470–86. 34958152 10.1111/jora.12711

[26] Carmo AS, Influence of parental perceived environment on physical activity, TV viewing, active play and body mass index among Portuguese children: a mediation analysis. Am J Hum Biol. 2020;32(6):e23400. 32027073 10.1002/ajhb.23400

[27] Kretschmer D, Kruse H. Neighbourhood effects on acculturation attitudes among minority and majority adolescents in Germany. Urban Stud. 2020;57(16):3363–80. 10.1177/0042098019897890.

[28] Machado-Rodrigues AM, Parental perceptions of neighborhood environments, BMI, and active behaviors in girls aged 7–9 years. Am J Hum Biol. 2014;26(5):670–5. 24943355 10.1002/ajhb.22577

[29] Pasco MC, White RMB, Iida M, Seaton EK. A prospective examination of neighborhood social and cultural cohesion and parenting processes on ethnic-racial identity among U.S. Mexican adolescents. Dev Psychol. 2021;57(5):783–95. 10.1037/dev0001170. 34166021 PMC8245012

[30] Spencer MB. “Phenomenology and ecological systems theory: development of diverse groups.” In: Handbook of Child Psychology. 2007.

[31] Curci SG, Luecken LJ, Perez M, White RM. Prenatal neighborhood ethnocultural context and the mental health of mothers and children in low-income Mexican American families. Child Dev. 2021;92(5):1785–800. 33929046 10.1111/cdev.13570 PMC11110471

[32] Browning CR, Dirlam J, Boettner B. From heterogeneity to concentration: Latino immigrant neighborhoods and collective efficacy perceptions in Los Angeles and Chicago. Soc Forces. 2016;95(2):779–807. 29430065 10.1093/sf/sow064 PMC5805395

[33] Budiman A and Ruiz NG. “Asian Americans are the fastest-growing racial or ethnic group in the U.S,” Pew Research Center, Washington D.C., 2021. [Online]. Available: https://www.pewresearch.org/fact-tank/2021/04/09/asian-americans-are-the-fastest-growing-racial-or-ethnic-group-in-the-u-s/. Accessed 7 Mar 2023.

[34] Office of Minority Health Resource Center U.S. Department of Health and Human Services, “Profile: Hispanic/Latino Americans,” https://minorityhealth.hhs.gov. Accessed 4 Apr 2023.

[35] Schneiderman N, Ironson G, Siegel SD. Stress and health: psychological, behavioral, and biological determinants. Annu Rev Clin Psychol. 2005;1:607–28. 10.1146/annurev.clinpsy.1.102803.144141. 17716101 PMC2568977

[36] Harville EW, Shankar A, DunkelSchetter C, Lichtveld M. Cumulative effects of the Gulf oil spill and other disasters on mental health among reproductive-aged women: the Gulf resilience on women’s health study. Psychol Trauma Theory Res Pract Policy. 2018;10:533–41. 10.1037/tra0000345. PMC10948975 29199841

[37] Los Angeles County Public Health. “Racial disparities in selected maternal characteristics and perinatal health indicators 2014 & 2016.” [Online]. Available: http://www.publichealth.lacounty.gov/mch/LAMB/Results/SPA%20indicator%20reports/SPA1_INDICATORS_FINAL.pdf. Accessed 10 Mar 2023.

[38] Aguilar-Gaxiola S, Loera G, Méndez L, Sala M, Latino Mental Health Concilio, Nakamoto J. “Community-defined solutions for Latino mental health care disparities: California reducing disparities project,” Latino strategic planning workgroup population report. Sacramento, CA: UC Davis, 2012. Available: https://health.ucdavis.edu/newsroom/pdf/latino_disparities.pdf. Accessed 4 Apr 2023.

[39] Alegría M, Mental health care for Latinos: inequalities in use of specialty mental health services among Latinos, African Americans, and Non-Latino Whites. Psychiatr Serv. 2002;53(12):1547–55. 10.1176/appi.ps.53.12.1547. 12461214

[40] Sue S, Zane N, Nagayama Hall GC, Berger LK. The case for cultural competency in psychotherapeutic interventions. Annu Rev Psychol. 2009;60:525–48. 10.1146/annurev.psych.60.110707.163651. 18729724 PMC2793275

[41] U.S. Department of Health and Human Services, Substance Abuse and Mental Health Services Administration, and Center for Behavioral Health Statistics and Quality. “National survey on drug use and health 2016 (NSDUH-2016-DS0001),” 2018. [Online]. Available: Retrieved from https://datafiles.samhsa.gov/. Accessed 7 Mar 2023.

[42] Centers for Disease Control and Prevention. “Summary health statistics: National Health Interview Survey: 2018. Table A-8.,” 2021. [Online]. Available: https://www.cdc.gov/nchs/nhis/shs/tables.htm.

[43] Wassertheil-Smoller S, Depression, anxiety, antidepressant use, and cardiovascular disease among Hispanic men and women of different national backgrounds: results from the Hispanic Community Health Study/Study of Latinos. Ann Epidemiol. 2014;24(11):822–30. 10.1016/j.annepidem.2014.09.003. 25439033 PMC4281495

[44] Huang ZJ, Wong FY, Ronzio CR, Yu SM. Depressive symptomatology and mental health help-seeking patterns of US-and foreign-born mothers. Matern Child Health J. 2007;11:257–67. 17171544 10.1007/s10995-006-0168-x

[45] U.S. Census Bureau. “QuickFacts Orange County, California,” 2021. [Online]. Available: https://www.census.gov/quickfacts/fact/dashboard/orangecountycalifornia/PST045221. Accessed 7 Mar 2023.

[46] Los Angeles County Commission for Women. “2016 report on the status of women in Los Angeles County,” 2016. [Online]. Available: http://laccw.lacounty.gov/LinkClick.aspx?fileticket=iZfSTtIdj4c%3D&portalid=10. Accessed 10 Mar 2023.

[47] Sampson RJ. “How do communities undergird or undermine human development? Relevant contexts and social mechanisms,” In: Does it take a village? Community effects on children, adolescents, and families, Psychology Press 2001;3–30.

[48] Sampson RJ, Raudenbush SW, Earls F. Neighborhoods and violent crime: a multilevel study of collective efficacy. Science. 1997;277(5328):918–24. 9252316 10.1126/science.277.5328.918

[49] White RM, Pasco MC, Korous KM, Causadias JM. A systematic review and meta-analysis of the association of neighborhood ethnic-racial concentrations and adolescent behaviour problems in the US. J Adolesc. 2020;78:73–84. 31869739 10.1016/j.adolescence.2019.12.005

[50] Jackson AL, Browning CR, Krivo LJ, Kwan M-P, Washington HM. The role of immigrant concentration within and beyond residential neighborhoods in adolescent alcohol use. J Youth Adolesc. 2016;45:17–34. 26215378 10.1007/s10964-015-0333-x PMC5476366

[51] Sampson RJ, Morenoff JD, Gannon-Rowley T. Assessing ‘neighborhood effects’: social processes and new directions in research. Annu Rev Sociol. 2002;28(1):443–78.

[52] White RM, Zeiders KH, Safa MD. Neighborhood structural characteristics and Mexican-origin adolescents’ development. Dev Psychopathol. 2018;30(5):1679–98. 30289093 10.1017/S0954579418001177

[53] Hayes-Bautista DE, Office of management and budget racial/ethnic categories in mortality research: a framework for including the voices of racialized communities. Am J Public Health. 2021;111(S2):S133–40. 10.2105/AJPH.2021.306361. 34314200 PMC8495649

[54] Kubrin CE, Weitzer R. New directions in social disorganization theory. J Res Crime Delinquency. 2003;40(4):374–402. 10.1177/0022427803256238.

[55] Vega WA, Ang A, Rodriguez MA, Finch BK. Neighborhood protective effects on depression in Latinos. Am J Community Psychol. 2011;47:114–26. 21052825 10.1007/s10464-010-9370-5

[56] Gonzales NA, Economic hardship, neighborhood context, and parenting: prospective effects on Mexican-American adolescent’s mental health. Am J Community Psychol. 2011;47(1–2):98–113. 10.1007/s10464-010-9366-1. 21103925 PMC3169846

[57] Campos B, Schetter CD, Abdou CM, Hobel CJ, Glynn LM, Sandman CA. Familialism, social support, and stress: positive implications for pregnant Latinas. Cultur Divers Ethnic Minor Psychol. 2008;14:155–62. 10.1037/1099-9809.14.2.155. 18426288 PMC2859297

[58] Nair RL, White RMB, Roosa MW, Zeiders KH. Cultural stressors and mental health symptoms among Mexican Americans: a prospective study examining the impact of the family and neighborhood context. J Youth Adolesc. 2013;42(10):1611–23. 10.1007/s10964-012-9834-z. 23111841 PMC3572237

[59] Cox JL, Holden JM, Sagovsky R. Detection of postnatal depression: development of the 10-item Edinburgh postnatal depression scale. Br J Psychiatry. 1987;150(6):782–6. 10.1192/bjp.150.6.782. 3651732

[60] Murray D, Cox JL. Screening for depression during pregnancy with the Edinburgh depression scale (EDDS). J Reprod Infant Psychol. 1990;8(2):99–107. 10.1080/02646839008403615.

[61] Marteau TM, Bekker H. The development of a six-item short-form of the state scale of the Spielberger State—Trait Anxiety Inventory (STAI). Br J Clin Psychol. 1992;31(3):301–6. 10.1111/j.2044-8260.1992.tb00997.x. 1393159

[62] Rini CK, Dunkel-Schetter C, Wadhwa PD, Sandman CA. Psychological adaptation and birth outcomes: the role of personal resources, stress, and sociocultural context in pregnancy. Health Psychol. 1999;18:333–45. 10.1037/0278-6133.18.4.333. 10431934

[63] Wadhwa PD, Sandman CA, Porto M, Dunkel-Schetter C, Garite TJ. The association between prenatal stress and infant birth weight and gestational age at birth: a prospective investigation. Am J Obstet Gynecol. 1993;169(4):858–65. 10.1016/0002-9378(93)90016-C. 8238139

[64] Bowins B Depression: discrete or continuous? Psychopathology. 2015;48(2):69–78. 10.1159/000366504. 25531962

[65] Hirschfeld RMA. The comorbidity of major depression and anxiety disorders: recognition and management in primary care. Prim Care Companion J Clin Psychiatry. 2001;3(6):244–54. 10.4088/pcc.v03n0609. 15014592 PMC181193

[66] Fox M Discrimination as a moderator of the effects of acculturation and cultural values on mental health among pregnant and postpartum Latina women. Am Anthropol. 2021;123(4):780–804. 10.1111/aman.13665. 36776224 PMC9909989

[67] Adler NE, Epel ES, Castellazzo G, Ickovics JR. “Relationship of subjective and objective social status with psychological and physiological functioning: preliminary data in healthy white women.” Health Psychol Off J Div Health Psychol Am Psychol Assoc. 2000;19(6):586–92. 10.1037//0278-6133.19.6.586. 11129362

[68] Hager ER, Development and validity of a 2-item screen to identify families at risk for food insecurity. Pediatrics. 2010;126(1):e26–32. 10.1542/peds.2009-3146. 20595453

[69] Freeman A, The role of socio-economic status in depression: results from the COURAGE (aging survey in Europe). BMC Public Health. 2016;16(1):1098. 10.1186/s12889-016-3638-0. 27760538 PMC5069819

[70] Perez LG, Ruiz JM, Berrigan D. Neighborhood environment perceptions among Latinos in the U.S. Int J Environ Res Public Health. 2019;16(17):17. 10.3390/ijerph16173062. PMC6747377 31443609

[71] van Buuren S, Groothuis-Oudshoorn K. mice: multivariate imputation by chained equations in R. J Stat Softw. 2011;45:1–67.

[72] Hunt MO, Wise LA, Jipguep M-C, Cozier YC, Rosenberg L. Neighborhood racial composition and perceptions of racial discrimination: evidence from the Black women’s health study. Soc Psychol Q. 2007;70(3):272–89. 10.1177/019027250707000306.

[73] White MP, Spending at least 120 minutes a week in nature is associated with good health and wellbeing. Sci Rep. 2019;9(1):1. 10.1038/s41598-019-44097-3. 31197192 PMC6565732

[74] Subiza-Pérez M, Does the perceived neighborhood environment promote mental health during pregnancy? Confirmation of a pathway through social cohesion in two Spanish samples. Environ Res. 2021;197:111192. 10.1016/j.envres.2021.111192. 33872648

[75] Schwartz SJ, Unger JB, Zamboanga BL, Szapocznik J. Rethinking the concept of acculturation: implications for theory and research. Am Psychol. 2010;65:237–51. 10.1037/a0019330. 20455618 PMC3700543

[76] van der Meer T, Tolsma J. Ethnic diversity and its effects on social cohesion. Annu Rev Sociol. 2014;40:459–78. 10.1146/annurev-soc-071913-043309.

[77] Thayer Z, Uwizeye G, McKerracher L. Toolkit article: approaches to measuring social inequities in health in human biology research. Am J Hum Biol. 2022;34(12):e23804. 10.1002/ajhb.23804. 36173013

[78] Grös DF, Antony MM, Simms LJ, McCabe RE. Psychometric properties of the State-Trait Inventory for Cognitive and Somatic Anxiety (STICSA): comparison to the State-Trait Anxiety Inventory (STAI). Psychol Assess. 2007;19:369–81. 10.1037/1040-3590.19.4.369. 18085930

[79] Fox M, Thayer ZM, Wadhwa PD. Acculturation and health: the moderating role of sociocultural context: context moderates how acculturation affects health. Am Anthropol. 2017;119(3):405–21. 10.1111/aman.12867. 28966344 PMC5617140

[80] Vega WA, Alderete E, Kolody B, Aguilar-Gaxiola S. Illicit drug use among Mexicans and Mexican Americans in California: the effects of gender and acculturation. Addiction. 1998;93(12):1839–50. 9926572 10.1046/j.1360-0443.1998.931218399.x

[81] Grote NK, Bridge JA, Gavin AR, Melville JL, Iyengar S, Katon WJ. A meta-analysis of depression during pregnancy and the risk of preterm birth, low birth weight, and intrauterine growth restriction. Arch Gen Psychiatry. 2010;67(10):1012–24. 20921117 10.1001/archgenpsychiatry.2010.111 PMC3025772

[82] Callaghan WM, MacDorman MF, Rasmussen SA, Qin C, Lackritz EM. The contribution of preterm birth to infant mortality rates in the United States. Pediatrics. 2006;118(4):1566–73. 17015548 10.1542/peds.2006-0860

[83] Eshete A, Alemu A, Zerfu TA. Magnitude and risk of dying among low birth weight neonates in Rural Ethiopia: a community-based cross-sectional study. Int J Pediatr. 2019;2019:1. 10.1155/2019/9034952 PMC6541952 31223314

[84] Cohen J Statistical power analysis for the behavioral sciences, 2nd ed. Hillsdale, NJ: Academic Press, 2013.

