# Supplementary material for: What Do Your Neighbors Think About You? How Perceived Neighbor Attitudes Toward Latinos Influence Mental Health Among a Pregnant Latina Cohort
Source: J Racial Ethn Health Disparities. Author manuscript; Available in PMC 2024 Aug 1. (PMC10756922; doi:10.1007/s40615-023-01684-5)
Supplement: supplement [file NIHMS1937012-supplement-supplement.docx]

**Supplementary Materials**

**Methods**

***Measures***

The Neighborhood Attitudes Toward Latinos Scale (Nair et al., 2013), which consists of six items (e.g., “*People in this neighborhood appreciate Latino culture and people*”) and measured on a 5-point Likert scale (0 = Not at all true to 4 = Very true).

The Edinburgh Postnatal Depression Scale (EPDS; Cox et al., 1987; Murry & Cox, 1990) consists of 10 items measured on a 4-point scale. As stated in the manuscript, each item was anchored on a 1-4 point scale (1 - “No, never” and 4 - “Yes, most of the time”). See Supplemental Table 1 for the item breakdown for this scale.

### Supplemental Table 1: Item Breakdown of EPDS Scale

| **Reverse Coded** | **EPDS Scale Item Text** | |
| --- | --- | --- |
|  | **English** | **Spanish** |
|  | I have been able to laugh and see the funny side of things | He podido reír y ver el lado gracioso de las cosas |
|  | I have looked forward with enjoyment to things | He mirado al futuro con placer para hacer cosas |
| x | I have blamed myself unnecessarily when things went wrong | Me he culpado sin necesidad cuando las cosas marchaban mal |
|  | I have been anxious or worried for no good reason | He estado ansiosa y preocupada sin motivo |
| x | I have felt scared or panicky for no very good reason | He sentido miedo o pánico sin motivo alguno |
| x | Things have been getting on top of me | Las cosas me oprimen o agobian |
| x | I have been so unhappy that I have had difficulty sleeping | Me he sentido tan infeliz, que he tenido dificultad para dormir |
| x | I have felt sad or miserable | Me he sentido triste y desgraciada |
| x | I have been so unhappy that I have been crying | Me he sentido tan infeliz que he estado llorando |
| x | The thought of harming myself has occurred to me | He pensado en hacerme daño |

The Spielberger State-Trait Anxiety Inventory State scale (STAI; validated among pregnant women by Marteau & Bekker, 1992), consists of six items, three of which are reversed coded, and anchored on a 4-point scale (1 = Not at all to 4 = Very much) with items like, “*I am worried*;” overall ɑ = .81; ɑ^E^ =.83 and ɑ^S^=.77). See Supplemental Table 2 for the item breakdown for this scale.

### Supplemental Table 2: Item Breakdown of STAI-SF Scale

| **Reverse Coded** | **STAI-SF Item Text** | |
| --- | --- | --- |
|  | **English** | **Spanish** |
| x | I feel calm | Me siento calmada |
|  | I feel upset | Me siento disgustada |
| x | I feel content | Me siento contenta |
| x | I am relaxed | Estoy relajada |
|  | I am worried | Estoy preocupada |
|  | I am tense | Estoy tensa |

The Pregnancy-Related Anxiety scale (PRA; Rini et al., 1999; Wadwa et al., 1993) consists of 10 items assessing concerns regarding women’s pregnancy and their fetus’ health (e.g., “*I am concerned or worried about how the baby is growing and developing inside me*”). These items are measured on a 4-point Likert scale (1 = Not at all/Never to 4 = Very much/Almost all of the time). ​

### Supplemental Table 3: Item Breakdown of PRA Scale

| **Reverse Coded** | **Pregnancy-Related Anxiety Scale Item Text** | |
| --- | --- | --- |
|  | **English** | **Spanish** |
| X | I am confident about having a normal childbirth. | Estoy segura de tener un parto normal. |
| X | I think my labor and delivery will go normally. | Creo que mi parto irá normalmente. |
|  | I am fearful regarding the health of my baby. | Temo por la salud de mi bebé. |
|  | I am worried that the baby might not be normal. | Me preocupa que el bebé no sea normal. |
|  | I am afraid that I will be harmed during delivery. | Temo que voy a sufrir daños durante el parto. |
|  | I am concerned or worried about how the baby is growing and developing inside me. | Estoy preocupada acerca de cómo el bebé está creciendo y desarrollándose dentro de mí. |
|  | I am concerned or worried about losing the baby. | Estoy preocupada de perder él bebé. |
|  | I am concerned or worried about having a hard or difficult labor and delivery. | Me preocupa o temo de tener un parto duro o difícil. |
|  | I am concerned or worried about taking care of a new baby. | Me preocupa o temo de cuidar a un bebé recién nacido. |
|  | I am concerned or worried about developing medical problems during the pregnancy. | Me preocupa o temo que desarrolle problemas médicos durante el embarazo. |

***Mental Health Clinical Cut-Offs***

We calculate clinical cut-offs scores for depression for descriptive purposes. Scores greater than 10 on the EPDS indicate likelihood of at least minor clinical depression. Debate surrounding the depression cut-off among English speakers suggests a cut-off of 12 or 13 (Matthey et al., 2017). However, validation of the EPDS among Spanish-speaking women (Garcia Esteve et al., 2003) recommends a cut-off of 10 or 11; while validation among Japanese pregnant women (Usuda et al., 2017) recommends a cut-off of 9. Here, we follow Garcia Esteve and colleagues’ (2003) recommendation.

***Confirmatory Factor Analysis***

We conducted a confirmatory factor analysis using Mplus 8.3 (Muthén & Muthén, 2019) on the Neighborhood Attitudes Toward Latinos Scale (Nair et al., 2013) in order to determine whether the items load onto a single latent factor. While the model produced an adequate fit, χ^2^(5) = 10.79, *p* = .06; CFI = .97; TLI = .94, RMSEA = .08, the reverse-scored items did not significantly load onto the factor (Supplementary Table 1).

**Supplementary Table 1**

*Confirmatory factor analysis for Neighborhood Attitudes Toward Latinos scale*

| Variable | Estimate | *p -* value |
| --- | --- | --- |
| YNAPPLAT | .71 | < .001 |
| YNSHARELAT | .96 | < .001 |
| YNSUPLAT | .62 | < .001 |
| YNLATOUT_RE | -.06 | .42 |
| YNCRITLAT_RE | -.11 | .10 |

*Note*. YNAPPLAT = “People in this neighborhood appreciate Latino culture and people.” YNSHARELAT = “People in this neighborhood talk about or share Latino customs like food or holidays.” YNSUPLAT = “People in this neighborhood get involved in activities to support the Latino community.” YNLATOUT_RE = “People in this neighborhood want to keep other Latino families out of the neighborhood (reverse-coded).” YNCRITLAT_RE = “People in this neighborhood are critical of Latinos (reverse-coded).”

***Imputation***

We examined missingness through the Amelia package in R. After removing the nine women who did not report whether they were U.S.-born or not, the overall variable missingness across the sub-study dataset there was only around 4% missingness. Each individual variable ranged in missingness from 0% (e.g., Ethnic Social Relations subscale) to 10.8% (food security).

We added variables with no or lower missingness that would help the chain equations fill in potential values for missing scores – specifically, we added the Ethnic Social Relations subscale, Neighborhood Ethnic Structure as well as a multidimensional acculturation measure (i.e., Acculturation Rating Scale for Mexican-Americans; ARSMA-II). These variables were chosen due to association with the variables of interest – particularly, socio-cultural measurements of both the individual and their neighborhood.

We assume the data is missing at random. We compared missingness maps of depressed (5% total missingness) and not depressed women (3%) based on their EPDS score (Garcia Esteve et al., 2003) and tested the percent missingness between the two groups (depressed: *M* = 1.49, *SD* = 2.05, not depressed, *M* = 1.09, *SD* = 1.56; *t*(47) = 1.13, *p* = .26). We also tested the overall response rate between those who identified as foreign-born and U.S.-born (missingness 5% for foreign-born, *M* = 1.48, *SD* = 2.18; and 4% for U.S.-born *M* = 1.17, *SD* = 1.65; *t*(235.5) = 1.27, *p* = .21) and found nothing falsifying our missing at random assumption.

Diagnostic plots were used to determine if imputation was within range for all variables (Supplementary Figure 1).

**Supplementary Figure 1**

*Diagnostic plots testing multiple imputation chain equation*

**
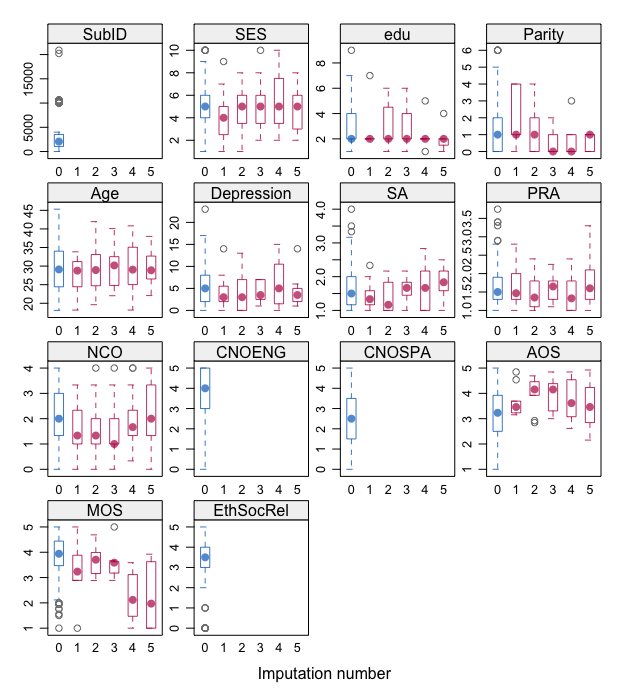
**

*Note.* SubID = Subject ID. SES = Socioeconomic Status. Edu = education status. Parity = parity. Age = age. Depression = depression. SA = state anxiety. PRA = pregnancy-related anxiety. NCO = Neighborhood Attitudes Toward Latinos Scale. CNOENG = Neighborhood Ethnic Structure - English. CNOSPA = Neighborhood Ethnic Structure – Spanish. AOS = Anglo orientation subscale of ARSMA-II. MOS = Mexican Orientation subscale of ARSMA-II. EthSocRel = Ethnic Social Relations subscale. Red dots indicate imputed variables, while blue dots indicate existing data. Some variables were either excluded from imputation (e.g., Subject IDs) or had no missing data to begin with (e.g., CNO).

***Regression Diagnostics***

The following supplemental table described relevant regression diagnostics for Model 1. Data were pooled for presentation in the main paper but presented individually here. Column 1 names the model. Column 2 describes the model's maximum variance inflation factor (VIF). The VIF measures whether there are high levels of multicollinearity. Variance-inflation factors (VIF) were calculated to examine correlations between independent variables to quantify multi-collinearity. While some scholars suggest a VIF of 5 or 10 to be problematic [85], we use the more conservative 2.5 limits [1], [2]. Some debate has circled around these cut-offs and their usefulness, but our results show that the VIF is consistently low indicating that multicollinearity is not a concern in our models. Column 3 presents the p-value results from the Breusch-Pagan test which looks for heteroskedasticity. Some models failed to reject the null hypothesis of heteroskedasticity (Supplementary Table 2). To account for heteroskedasticity and remain conservative in our estimates, robust standard errors were calculated for all models. Column 4 is the adjusted R^2^ for each model, which was calculated in order to describe how much variance of the outcome variable is associated with the predictors, including a penalty to account for the number of predictor variables. Residual plots run iteratively on each model for each imputed dataset did not reveal anything out of the ordinary (e.g., no non-linear relationships or high-leverage data points).

**Supplementary Table 2**

*Regression Diagnostics for Model Foreign Born*

| **Model** | **Maximum VIF** | **Breusch Pagan *p*-value** | **Adjusted R-squared** |
| --- | --- | --- | --- |
| Depression Imputation Model #1 | 1.28 | 0.00 | 0.43 |
| Depression Imputation Model #2 | 1.24 | 0.01 | 0.41 |
| Depression Imputation Model #3 | 1.26 | 0.00 | 0.40 |
| Depression Imputation Model #4 | 1.27 | 0.01 | 0.45 |
| Depression Imputation Model #5 | 1.24 | 0.01 | 0.38 |
| State Anxiety Imputation Model #1 | 1.31 | 0.12 | 0.42 |
| State Anxiety Imputation Model #2 | 1.23 | 0.7 | 0.38 |
| State Anxiety Imputation Model #3 | 1.28 | 0.27 | 0.39 |
| State Anxiety Imputation Model #4 | 1.33 | 0.03 | 0.42 |
| State Anxiety Imputation Model #5 | 1.20 | 0.02 | 0.38 |
| Pregnancy-Related Anxiety Imputation Model #1 | 1.84 | 0.01 | 0.18 |
| Pregnancy-Related Anxiety Imputation Model #2 | 1.76 | 0.01 | 0.15 |
| Pregnancy-Related Anxiety Imputation Model #3 | 1.75 | 0.00 | 0.17 |
| Pregnancy-Related Anxiety Imputation Model #4 | 1.88 | 0.00 | 0.16 |
| Pregnancy-Related Anxiety Imputation Model #5 | 1.68 | 0.00 | 0.16 |

*Note.* The unpooled, adjusted-R2 of the foreign-born women run iteratively on each imputed dataset while penalizing for the number of covariates in the model was 0.41 (min: 0.38, max 0.45) for depression, 0.4 (min: 0.38, max 0.42) for state anxiety, and 0.16 (min: 0.15, max 0.18) for pregnancy-related anxiety.

**Supplementary Table 3**

*Regression Diagnostics for Model U.S.-Born*

| **Model** | **Maximum VIF** | **Breusch Pagan *p*-value** | **Adjusted R-squared** |
| --- | --- | --- | --- |
| Depression Imputation Model #1 | 1.39 | 0.01 | 0.56 |
| Depression Imputation Model #2 | 1.39 | 0.01 | 0.57 |
| Depression Imputation Model #3 | 1.42 | 0.01 | 0.56 |
| Depression Imputation Model #4 | 1.36 | 0.01 | 0.58 |
| Depression Imputation Model #5 | 1.41 | 0.01 | 0.55 |
| State Anxiety Imputation Model #1 | 1.46 | 0.58 | 0.53 |
| State Anxiety Imputation Model #2 | 1.48 | 0.56 | 0.54 |
| State Anxiety Imputation Model #3 | 1.49 | 0.57 | 0.53 |
| State Anxiety Imputation Model #4 | 1.43 | 0.63 | 0.54 |
| State Anxiety Imputation Model #5 | 1.45 | 0.6 | 0.53 |
| Pregnancy-Related Anxiety Imputation Model #1 | 2.26 | 0.85 | 0.29 |
| Pregnancy-Related Anxiety Imputation Model #2 | 2.33 | 0.85 | 0.27 |
| Pregnancy-Related Anxiety Imputation Model #3 | 2.25 | 0.84 | 0.31 |
| Pregnancy-Related Anxiety Imputation Model #4 | 2.36 | 0.87 | 0.27 |
| Pregnancy-Related Anxiety Imputation Model #5 | 2.22 | 0.84 | 0.29 |

*Note:* The unpooled, adjusted-R2 of the model for US Born women run iteratively on each imputed dataset while penalizing for the number of covariates in the model among US-born women was 0.55 (min: 0.48, max 0.58) for depression, 0.52 (min: 0.45, max 0.54) for state anxiety, and 0.27 (min: 0.2, max 0.31) for pregnancy-related anxiety.

***Testing heterogeneity between U.S.-born and foreign-born groups***

Here we reproduce our Table 1 in the manuscript but with a new column 3 to assess the heterogeneity between the U.S.-born and foreign-born groups for each variable through t-tests for continuous variables and chi-square tests for categorical variables. The new third column is a list of p-values from these statistical tests, which reveals that our foreign-born and U.S.-born sub-cohorts only differ statistically in age and socio-economic status (the latter of which we control for in our models). The variables of interest, particularly neighborhood (as shown by the estimations of neighbor attitudes towards Latino’s **and** the self-reported ethnic composition of the neighborhood) and mental-health measures (depression and anxiety) do not statistically differ between these groups. This lack of significance supports the idea that we are likely presenting a unique finding from our models rather than only mapping differences between the two groups.

| **Supplementary Table 4**  *Cohort Demographics* | | | |
| --- | --- | --- | --- |
|  | **U.S. Born (N=108)** | **Foreign Born (N=131)** | **p-value** |
| **Age (years)** |  |  |  |
| Mean (SD) | 27.7 (5.51) | 30.9 (6.33) | <0.001 |
| Median [Min, Max] | 27.2 [18.2, 42.0] | 30.7 [18.1, 45.3] |  |
| Missing | 5 (4.6%) | 6 (4.6%) |  |
| **Relationship Status** |  |  |  |
| In a relationship | 94 (87.0%) | 116 (88.5%) | 0.807 |
| Single | 11 (10.2%) | 11 (8.4%) |  |
| Missing | 3 (2.8%) | 4 (3.1%) |  |
| **Parity** |  |  |  |
| Nulliparous | 43 (39.8%) | 41 (31.3%) | 0.305 |
| Parous | 26 (24.1%) | 37 (28.2%) |  |
| Missing | 39 (36.1%) | 53 (40.5%) |  |
| **Education** |  |  |  |
| Less than High School | 4 (3.7%) | 27 (20.6%) | <0.001 |
| High School or Equivalent | 67 (62.0%) | 55 (42.0%) |  |
| More than High School | 35 (32.4%) | 44 (33.6%) |  |
| Missing | 2 (1.9%) | 5 (3.8%) |  |
| **Trimester** |  |  |  |
| First Trimester | 10 (9.3%) | 8 (6.1%) | 0.673 |
| Second Trimester | 23 (21.3%) | 29 (22.1%) |  |
| Third Trimester | 65 (60.2%) | 80 (61.1%) |  |
| Missing | 10 (9.3%) | 14 (10.7%) |  |
| **Food Insecurity** |  |  |  |
| Yes | 41 (38.0%) | 53 (40.5%) | 0.81 |
| No | 55 (50.9%) | 64 (48.9%) |  |
| Missing | 12 (11.1%) | 14 (10.7%) |  |
| **Country of Origin** |  |  |  |
| U.S. | 108 (100%) | 0 (0%) | <0.001 |
| Mexico | 0 (0%) | 103 (78.6%) |  |
| El Salvador | 0 (0%) | 12 (9.2%) |  |
| Guatemala | 0 (0%) | 7 (5.3%) |  |
| Another country | 0 (0%) | 6 (4.6%) |  |
| Missing | 0 (0%) | 3 (2.3%) |  |
| **Depression (EPDS) (full scale range)** |  |  |  |
| Mean (SD) | 5.46 (4.71) | 5.70 (4.53) | 0.693 |
| Median [Min, Max] | 5.00 [0, 23.0] | 5.00 [0, 17.0] |  |
| Missing | 2 (1.9%) | 6 (4.6%) |  |
| **State Anxiety (full scale range)** |  |  |  |
| Mean (SD) | 1.73 (0.625) | 1.63 (0.534) | 0.203 |
| Median [Min, Max] | 1.67 [1.00, 4.00] | 1.50 [1.00, 3.17] |  |
| Missing | 3 (2.8%) | 8 (6.1%) |  |
| **Pregnancy-Related Anxiety (full scale range)** |  |  |  |
| Mean (SD) | 1.61 (0.529) | 1.67 (0.573) | 0.416 |
| Median [Min, Max] | 1.50 [1.00, 3.30] | 1.50 [1.00, 3.75] |  |
| Missing | 2 (1.9%) | 8 (6.1%) |  |
| **Number of Latino-identifying neighbors reported by participants** |  |  |  |
| About half the people | 33 (30.6%) | 29 (22.1%) | 0.127 |
| Everyone or almost everyone | 21 (19.4%) | 34 (26.0%) |  |
| Less than half the people | 12 (11.1%) | 23 (17.6%) |  |
| More than half the people | 29 (26.9%) | 24 (18.3%) |  |
| Nobody or almost nobody | 5 (4.6%) | 10 (7.6%) |  |
| Missing | 8 (7.4%) | 11 (8.4%) |  |
| **Neighborhood Attitudes Toward Latinos Scale** |  |  |  |
| Mean (SD) | 2.16 (1.18) | 2.27 (1.07) | 0.492 |
| Median [Min, Max] | 2.00 [0, 4.00] | 2.00 [0, 4.00] |  |
| Missing | 8 (7.4%) | 13 (9.9%) |  |

**References**

[1] R. Johnston, K. Jones, and D. Manley, “Confounding and collinearity in regression

analysis: a cautionary tale and an alternative procedure, illustrated by studies of British voting behaviour,” *Qual. Quant.*, vol. 52, no. 4, pp. 1957–1976, 2018, doi: 10.1007/s11135-017-0584-6.

[2] T. A. Craney and J. G. Surles, “Model-Dependent Variance Inflation Factor Cutoff

Values,” *Qual. Eng.*, vol. 14, no. 3, pp. 391–403, Mar. 2002, doi: 10.1081/QEN-120001878.
